# Fees for laboratory analyses of tobacco and related products in Europe: The next step forward

**DOI:** 10.18332/tpc/161896

**Published:** 2023-04-21

**Authors:** Enrico Davoli, Silvano Gallus, Federica Mattioli, Alessandra Lugo, Renata Solimini, Francisco R. Domínguez, Miguel M. Troasur, Constantine Vardavas

**Affiliations:** 1 Istituto di Ricerche Farmacologiche Mario Negri IRCCS, Milano, Italy; 2 Istituto Superiore di Sanità ( ISS), Rome, Italy; 3 Andalusian Regional Ministry of Health and Consumer Affairs, General Directorate of Public Health, Seville, Spain; 4 Agency for Agrarian and Fisheries Management of Andalusia, Seville, Spain; 5 University of Crete, Heraklion, Greece; * On behalf of the JATC WP4 partners

**Keywords:** Tobacco Products Directive, tobacco products, laboratory analysis

**Dear Editor,**

The EU Tobacco Products Directive (TPD) 2014/40/EU sets out measurement methods for ingredients and emissions levels for tobacco products (Articles 3 and 4) and regulates ingredients (Article 7)^1^. Laboratory measurements are essential for the effective application of various provisions of the TPD. In particular, the Competent Authorities (CA) of all the European Union (EU) Member States (MS) ‘shall communicate to the European Commission a list of approved laboratories, specifying the criteria used for approval and the methods of monitoring applied’ (Article 4). The laboratories should verify the tar, nicotine, and carbon monoxide (TNCO) emission levels of cigarettes. The TPD requires that these laboratories are independent and ‘shall not be owned or controlled directly or indirectly by the tobacco industry’.

Within the context of the Joint Action on Tobacco Control (JATC 1), a preliminary Competent Authority Survey was performed by the JATC work package (WP8) partners, across all the EU MS, with 24 respondents from 19 MS. Main findings of this survey (Figure 1) revealed that a relatively high proportion of CA did not request any verification analysis between December 2018 and January 2019; with the exception of two active CA with strong verification programs, and most CA requested only a limited number of analyses. The analyses requested were limited to cigarettes and, even less, to electronic cigarettes, while verifications of other products (heated tobacco products, oral tobacco, herbal tobacco, cigars, pipe) were negligible. Finally, a relatively high proportion of CA required verification from non-approved laboratories (also for TNCO).

**Figure 1:**
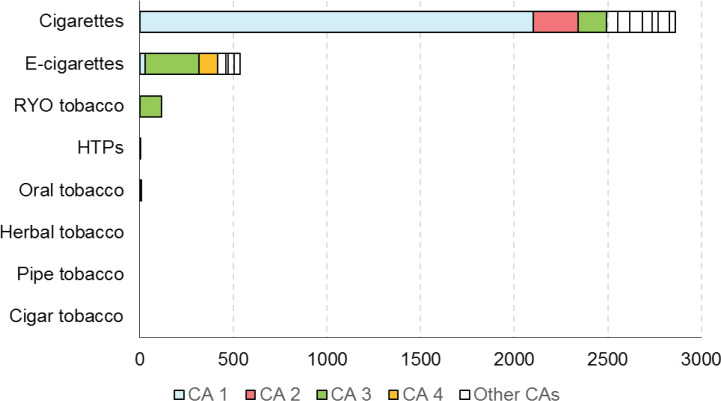
Number of analyses requested by Competent Authorities (CAs) for each tobacco product category^2^ in 2017–2018, as determined from a Competent Authority Survey conducted within JATC 1 across all EU Member States. The data were obtained from 24 respondents across 19 Member States

A laboratory survey^2^, based on the responses from 28 independent laboratories in 17 MS, indicated that the laboratories performed verification of parameters in 9 MS. Results indicated that for the analysis of cigarettes (TNCO) and electronic cigarettes/ heated tobacco products nicotine parameters, the methods used followed international standards with laboratories procedures that were accredited or validated. In e-liquids, nicotine, glycerol, and propylene glycol concentrations are the only parameters verified with standard procedures by CA accredited laboratories.

The above results produced within the context of the EU JATC indicate the need for enhanced collaboration between EU MS regarding the analysis in tobacco products, electronic cigarette liquids and heated tobacco products. Supporting collaboration across laboratories would be an efficient way to ensure laboratory capacity to verify new products and support enforcement.

According to the legal text of the TPD, MS may charge manufacturers and importers of tobacco products proportionate fees/retributions for the verification of (at least) TNCO emission levels. Most of the analyses conducted in 2017–2018, however, were not paid by the tobacco industry and all the respondents of the conducted survey recognized consistently a lack of financial support for the verification. For this reason, 70% of the CA from the JATC WP8 survey, believed that the fees for the verification analysis of cigarettes, electronic cigarettes and other tobacco products should be fully or partially covered by manufacturers.

For the coverage of the costs for Independent laboratories to test tobacco products (including electronic cigarettes and e-liquids), we support the recommendations by WHO^3,4^ that tobacco manufacturers should bear all testing costs. The introduction of a fee (contribution to the costs) for laboratory tests (Fee for Tobacco Laboratories) will be fundamental to support the legislation and harmonization process of tobacco products verification on EU level^4^. This fee, or proportionate retribution, should support independent laboratories through governmental bodies (particularly the Ministry of Finance and Ministry of Health), to increase the number of verification programs, the number of MS with approved laboratories and to expand collaborations among laboratories, an aspect that will be addressed within the JATC 2 project^5^.

By filling this collaboration gap on aspects of laboratory measurement, the TPD poses an important and effective step in the regulation of tobacco ingredients and emissions, with the ultimate aim to protect public health in Europe.

## Data Availability

Data sharing is not applicable to this article as no new data were created.
